# Association of Hydrochlorothiazide Use and Risk of Malignant Melanoma

**DOI:** 10.1001/jamainternmed.2018.1652

**Published:** 2018-05-29

**Authors:** Anton Pottegård, Sidsel Arnspang Pedersen, Sigrun Alba Johannesdottir Schmidt, Lisbet Rosenkrantz Hölmich, Søren Friis, David Gaist

**Affiliations:** 1 Clinical Pharmacology and Pharmacy, Department of Public Health, University of Southern Denmark, Odense, Denmark; 2 Department of Neurology, Odense University Hospital, Odense, Denmark; 3 Department of Clinical Research, Faculty of Health Sciences, University of Southern Denmark, Odense, Denmark; 4 Department of Clinical Epidemiology, Aarhus University Hospital, Aarhus, Denmark; 5 Department of Plastic Surgery, Herlev Gentofte Hospital, Herlev, Denmark; 6 Danish Cancer Society Research Center, Danish Cancer Society, Copenhagen, Denmark; 7 Department of Public Health, University of Copenhagen, Denmark

## Abstract

This case-control study assesses the association of hydrochlorothiazide with risk of malignant melanoma among Danish adults.

We have recently shown that hydrochlorothiazide use increases the risk of lip and nonmelanoma skin cancer, notably squamous cell carcinoma.^1,2^ It would have substantial implications if the carcinogenic effect of hydrochlorothiazide also extended to malignant melanoma.

## Methods

Similarly to our recent studies of hydrochlorothiazide,^1,2^ we identified histologically verified melanoma cases (January 2004 to December 2015), each matched 1:10 (risk-set sampling; age, sex, and date) to cancer-free population controls. We required cases and controls to be between ages 18 and 90 years, without previous history of cancer (except nonmelanoma skin cancer), organ transplantation, HIV infection, or azathioprine use, and to have resided continuously in Denmark for 10 years.

Using conditional logistic regression, we calculated odds ratios (ORs), with 95% CIs, for melanoma associated with cumulative hydrochlorothiazide use compared with never-use, adjusting for potential confounders (Table 1 and 2). We performed stratified analyses by localization, stage, histologic subtype, and subgroups of age, sex, and history of nonmelanoma skin cancer. To evaluate potential confounding by indication, we performed analyses for other antihypertensive drugs, including bendroflumethiazide, angiotensin-converting enzyme inhibitors, angiontensin-II receptor antagonists, and calcium-channel blockers. This study was approved by Statistics Denmark and the Danish Data Protection Agency.

**Table 1.** Association of Exposure to Hydrochlorothiazide and Risk of Malignant Melanoma

Use and Dose	Cases, No. (n = 19 273)	Controls, No. (n = 192 730)	Adjusted OR (95% CI)	
			Model 1^a^	Model 2^b^
**Hydrochlorothiazide**				
Never used	17 315	175 486	1 [Reference]	1 [Reference]
Ever used	1958	17 244	1.16 (1.11-1.22)	1.17 (1.11-1.23)
High use (≥50 000 mg)	413	3406	1.24 (1.12-1.38)	1.22 (1.09-1.36)
**Cumulative dose**				
1-24 999 mg	1160	10 483	1.14 (1.07-1.21)	1.14 (1.07-1.22)
25 000-49 999 mg	385	3355	1.18 (1.06-1.32)	1.18 (1.05-1.32)
50 000-99 999 mg	219	1852	1.22 (1.06-1.41)	1.21 (1.05-1.40)
≥100 000 mg	194	1554	1.26 (1.08-1.46)	1.21 (1.04-1.42)
Test for trend	1958	17 244	*P* = .24	*P* = .42

^a^ Adjusted for age, sex, and calendar time (by use of risk-set matching and conditional analysis).

^b^ Fully adjusted model additionally adjusted for history of nonmelanoma skin cancer, other comorbidity (diabetes, chronic obstructive pulmonary disease, alcohol abuse–associated disorders, chronic renal failure), Charlson Comorbidity Index score (0, low; 1-2, medium; or ≥3, high), use of certain drugs (topical retinoids, oral retinoids, tetracycline, macrolides, aminoquinolines, amiodarone, methoxypsoralen, low-dose aspirin, nonaspirin nonsteroidal anti-inflammatory drugs, statins, or oral steroids), and highest achieved education (short, medium, long, or unknown).

**Table 2.** Association of Exposure to Hydrochlorothiazide and Risk of Malignant Melanoma According to Amount of Hydrochlorothiazide Use and Specified by Melanoma Subtype

Melanoma	Cases. No.	Controls, No.	Adjusted OR (95% CI)	
			Model 1^a^	Model 2^b^
**Superficial Spreading Melanoma**				
Never used	12 494	126 216	1 [Reference]	1 [Reference]
Ever used	1287	11 594	1.13 (1.06-1.20)	1.13 (1.06-1.20)
High use (≥50 000 mg)	254	2268	1.14 (0.99-1.30)	1.11 (0.97-1.27)
Cumulative dose				
1-24 999 mg	783	7023	1.14 (1.05-1.23)	1.13 (1.05-1.23)
25 000-49 999 mg	250	2303	1.11 (0.97-1.27)	1.10 (0.96-1.27)
50 000-99 999 mg	140	1252	1.15 (0.96-1.38)	1.14 (0.95-1.37)
≥100 000 mg	114	1016	1.12 (0.91-1.36)	1.06 (0.87-1.30)
Test for trend	1287	11 594	*P* = .94	*P* = .73
**Nodular Melanoma**				
Never used	1465	15 108	1 [Reference]	1 [Reference]
Ever used	230	1842	1.31 (1.12-1.53)	1.28 (1.09-1.49)
High use (≥50 000 mg)	68	351	2.13 (1.61-2.80)	2.05 (1.54-2.72)
Cumulative dose				
1-24 999 mg	119	1142	1.08 (0.88-1.32)	1.05 (0.86-1.29)
25 000-49 999 mg	43	349	1.24 (0.90-1.72)	1.17 (0.84-1.64)
50 000-99 999 mg	34	195	1.90 (1.30-2.78)	1.81 (1.23-2.67)
≥100 000 mg	34	156	2.34 (1.59-3.45)	2.26 (1.52-3.36)
Test for trend	230	1842	*P* = .01	*P* = .01
**Lentigo Melanoma**				
Never used	386	4198	1 [Reference]	1 [Reference]
Ever used	114	802	1.57 (1.25-1.97)	1.58 (1.25-2.00)
High use (≥50 000 mg)	28	177	1.72 (1.13-2.62)	1.61 (1.03-2.50)
Cumulative dose				
1-24 999 mg	58	476	1.32 (0.98-1.78)	1.35 (0.99-1.83)
25 000-49 999 mg	28	149	2.22 (1.44-3.43)	2.30 (1.46-3.60)
50 000-99 999 mg	11	99	1.25 (0.66-2.38)	1.09 (0.56-2.11)
≥100 000 mg	17	78	2.26 (1.30-3.91)	2.24 (1.25-3.99)
Test for trend	114	802	*P* = .11	*P* = .16

^a^ Adjusted for age, sex, and calendar time (by use of risk-set matching and conditional analysis).

^b^ Fully adjusted model additionally adjusted for history of nonmelanoma skin cancer, other comorbidity (diabetes, chronic obstructive pulmonary disease, alcohol abuse associated disorders, chronic renal failure), Charlson Comorbidity Index score (0, low; 1-2, medium; or ≥3, high), use of certain drugs (topical retinoids, oral retinoids, tetracycline, macrolides, aminoquinolines, amiodarone, methoxypsoralen, low-dose aspirin, nonaspirin nonsteroidal anti-inflammatory drugs, statins, or oral steroids), and highest achieved education (short, medium, long, or unknown).

## Results

We identified 22 010 cases of melanoma. After exclusions, the final study population comprised 19 273 cases and 192 730 population controls. Cases had slightly lower comorbidity, higher educational level, and higher prevalence of previous nonmelanoma skin cancer than controls. Remaining characteristics were similar between cases and controls.

Overall, 413 cases (2.1%) and 3406 controls (1.8%) were classified as high-users (≥50 000 mg) of hydrochlorothiazide, yielding an adjusted OR of 1.22 (95% CI, 1.09-1.36) for melanoma. No clear dose-response pattern emerged between hydrochlorothiazide use and melanoma risk (Table 1). Analyses by melanoma localization, stage, age, sex, and history of nonmelanoma skin cancer yielded results comparable to the main analysis (data not shown). When stratifying by histological subtype (Table 2), higher ORs occurred for nodular melanoma (n = 1695 cases [8.8%]; OR, 2.05; 95% CI, 1.54-2.72; *P* for trend = .01) and lentigo melanoma (n = 500 cases [2.6%]; OR, 1.61; 95% CI, 1.03-2.50; *P* for trend = .16) than for superficial spreading melanoma (n = 13 781 cases [72%]; OR, 1.11; 95% CI, 0.97-1.27; *P* for trend = .73).

In secondary analyses, we observed associations close to the null for overall melanoma risk with long-term use of bendroflumethiazide (OR, 1.10; 95% CI, 1.02-1.19; *P *for trend = 0.47), angiotensin-converting enzyme inhibitors (OR, 1.07; 95% CI, 0.99-1.16; *P *for trend = .53), angiotensin-II receptor antagonists (OR, 1.18; 95% CI, 1.07-1.29; *P *for trend = .07), and calcium-channel blockers (OR, 1.06; 95% CI, 0.97-1.14; *P *for trend  = .94). These associations remained neutral in subanalyses stratified by melanoma subtype (data not shown).

## Discussion

The main strength of our study is the use of high-quality nationwide registry data.^3^ The main limitations are a lack of information on risk factors such as sun exposure, skin pigmentation, and family history of melanoma. However, these characteristics are unlikely to be substantially associated with hydrochlorothiazide use, and thus unlikely to confound out estimates.

Thiazide use and melanoma risk has been investigated in a few previous studies; however, only 2 studies,^4,5^ both from northern Denmark, have specifically examined hydrochlorothiazide. The first study reported an OR of 1.32 (95% CI, 1.03-1.70) for melanoma risk overall associated with 10 000 mg increments of hydrochlorothiazide.^4^ The corresponding OR for hydrochlorothiazide in combination with amiloride was 1.43 (95% CI, 1.09-1.88).^4^ The other study found no association between hydrochlorothiazide use combined with amiloride and melanoma risk (OR, 1.02; 95% CI, 0.78-1.33).^5^ Neither of these studies included dose-response or histology-specific analyses.

The findings for melanoma subtype are somewhat surprising, as lentigo and superficial spreading melanoma are known to be associated with high sun exposure, whereas the etiology of nodular melanomas is less elucidated.^6^ It is worrying that hydrochlorothiazide use appears to be associated with an increased risk of melanoma, and the particular associations observed for lentigo melanoma and nodular melanoma warrant further research.

## References

[1] Pottegård A, Hallas J, Olesen M, Hydrochlorothiazide use is strongly associated with risk of lip cancer. J Intern Med. 2017;282(4):322-331. doi:10.1111/joim.12629 28480532

[2] Arnspang S, Gaist D, Johannesdottir Schmidt SA, Hölmich LR, Friis S, Pottegård A Hydrochlorothiazide use and risk of non-melanoma skin cancer: A nationwide case-control study from Denmark. J Am Acad Dermatol. 2018;78(4):673-581.e9. doi:10.1016/j.jaad.2017.11.042 29217346

[3] Schmidt M, Pedersen L, Sørensen HT The Danish Civil Registration System as a tool in epidemiology. Eur J Epidemiol. 2014;29(8):541-549. doi:10.1007/s10654-014-9930-3 24965263

[4] Jensen AØ, Thomsen HF, Engebjerg MC, Olesen AB, Sørensen HT, Karagas MR Use of photosensitising diuretics and risk of skin cancer: a population-based case-control study. Br J Cancer. 2008;99(9):1522-1528. doi:10.1038/sj.bjc.6604686 18813314 PMC2579687

[5] Schmidt SAJ, Schmidt M, Mehnert F, Lemeshow S, Sørensen HT Use of antihypertensive drugs and risk of skin cancer. J Eur Acad Dermatol Venereol. 2015;29(8):1545-1554. doi:10.1111/jdv.12921 25640031

[6] Whiteman DC, Stickley M, Watt P, Hughes MC, Davis MB, Green AC Anatomic site, sun exposure, and risk of cutaneous melanoma. J Clin Oncol. 2006;24(19):3172-3177. doi:10.1200/JCO.2006.06.1325 16809740

